# Intra-articular fibrous bands at the tibiotalar joint: diagnosis and outcomes of arthroscopic removal in 4 ankles

**DOI:** 10.1186/s40634-021-00360-z

**Published:** 2021-06-23

**Authors:** Philippe Beaudet, Floris van Rooij, Mo Saffarini, Alexis Nogier

**Affiliations:** 1 Clinique Trénel, 575 Rue Trénel, 69560 Sainte-Colombe, France; 2 ReSurg SA, Rue Saint Jean 22, 1260 Nyon, Switzerland

## Abstract

The authors retrieved the records of 4 patients that exhibited unusual structural anomalies or pathologies, notably the presence of a fibrous band at the anterior aspect of the tibiotalar joint, observed during arthroscopic exploration or treatment between January and December 2019. Only 1 patient had surgical antecedents on the ipsilateral ankle (extra-articular tenodesis 10 years earlier). The remaining 3 patients had no surgical antecedents on the ipsilateral ankle. The fibrous band was removed in all patients during arthroscopic Brostöm procedure or exploration. For the first 3 patients, the intra-articular fibrous band was not observed prior to arthroscopy by either the senior surgeon or radiologist on any of the images (2 MRIs and 1 CTA), but retrospective inspection confirmed that the intra-articular fibrous band was present but had been overlooked.

At a follow-up of 22.3 ± 5.0 months (range, 15–26), all patients reported a decrease in pVAS (− 5.0 ± 2.6, range, 2–8), and an improvement in AOFAS (51.0 ± 17.7, range, 26–65), EFAS (14.5 ± 8.7, range, 6–23) and EFAS sport (8.0 ± 5.3, range, 2–10).

This case report corroborates the findings of an earlier discovery of an intra-articular fibrous band in 4 ankles, with more detailed information for clinical and radiologic diagnosis, as well as outcomes of arthroscopic removal. Clinicians should beware of such foreign bodies in the ankle, particularly in patients with history of sprains, and consider arthroscopic removal in cases with persistent pain and/or functional impairment.

## Introduction

A “web-like intra-articular fibrous band” in the ankle joint has been recently described [1, 9, 12, 14]. Valkering et al. [14], reported on 2 cases with post-traumatic anterior ankle pain. Arthroscopic removal of this fibrous band relieved pain and restored mobility. The etiology of this intra-articular fibrous band is not yet known, but it may be related to bleeding after trauma within the anterior ankle chamber, which can create fibrous structures in the joint [5].

As the discovery of this fibrous band is recent, there is little information on its diagnosis, symptoms and treatment options. The aim of this case report is to facilitate the diagnosis of intra-articular fibrous bands using 3D imaging, and report outcomes of their arthroscopic removal in 4 ankles.

## Case report

**Fig. 1:**
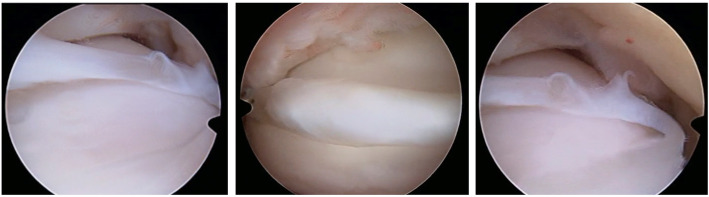
Arthroscopic images of the fibrous band

Between January and December 2019, the senior author performed arthroscopic procedures on 62 ankles, 4 (6.5%) of which exhibited unusual structural anomalies or pathologies, notably the presence of a fibrous band at the anterior aspect of the tibiotalar joint (Fig. 1). The patients comprised 2 men and 2 women, aged 33.8 ± 11.6 (range, 24–50), with a body mass index (BMI) of 25.0 ± 4.8 (range, 19–29) at the time of arthroscopy (Table 1). Three patients had sustained ankle sprains within the previous 2 years (between August 2017 and July 2018) but had no surgical antecedents on the ipsilateral ankle, whereas one patient had sustained an ankle sprain 12 years beforehand (April 2008), which was treated by extra-articular tenodesis 10 years beforehand (December 2009) (Appendix 1).

**Table 1:** Demographics and clinical assessment

	Patient 1	Patient 2	Patient 3	Patient 4	Mean ± SD All patients
**Sex**	Female	Female	Male	Male	
**Age**	27	24	34	50	33.8 ± 11.6
**BMI**	24	19	29	28	25.0 ± 4.8
**Work accident**	No	No	Yes	Yes	
**Time between injury and surgery** *(years)*	0.67	1.58	0.50	11.67	3.6 ± 5.4
**Broström procedure**	No	Yes	Yes	No	
**Osteochondral lesion**	No	Yes	No	No	
**Follow-up** *(months)*	24	24	26	15	22.3 ± 5.0
**pVAS** (10–0)					
preoperative	7	8	8	4	6.8 ± 1.9
postoperative	1	0	4	2	1.8 ± 1.7
net improvement	−6	−8	−4	−2	−5.0 ± 2.6
**AOFAS** (0–100)					
preoperative	35	23	23	50	32.8 ± 12.8
postoperative	97	88	74	76	83.8 ± 10.8
net improvement	62	65	51	26	51.0 ± 17.7
**EFAS** (0–24)					
preoperative	0	1	5	4	2.5 ± 2.4
postoperative	23	22	11	12	17.0 ± 6.4
net improvement	23	21	6	8	14.5 ± 8.7
**EFAS sport** (0–16)					
preoperative	0	0	n/a	1	0.3 ± 0.6
postoperative	12	10	n/a	3	8.3 ± 4.7
net improvement	12	10	n/a	2	8.0 ± 5.3
**ROM (°)**					
preoperative	40	60	60	35	48.8 ± 13.2
postoperative	60	60	60	45	56.3 ± 7.5
net improvement	20	0	0	10	7.5 ± 9.6

Slice images of the ankle had been acquired 1 to 7 months prior to arthroscopy: 3 magnetic resonance images (MRI) and 1 computed tomography arthrography (CTA). The slice images revealed thickening of the anterior talofibular ligament (ATFL) in 2 ankles (MRI), and a synovial fragment in 1 ankle (MRI). The intra-articular fibrous band were not noticed on any of the images taken prior to arthroscopy (3 MRIs and 1 CTA), by either the senior surgeon or radiologist, but retrospective inspection of the images confirmed that intra-articular fibrous bands were present in all 4 patients prior to arthroscopy (including on the MRI taken in the patient that had extra-articular tenodesis 10 years beforehand).

**Fig. 2:**
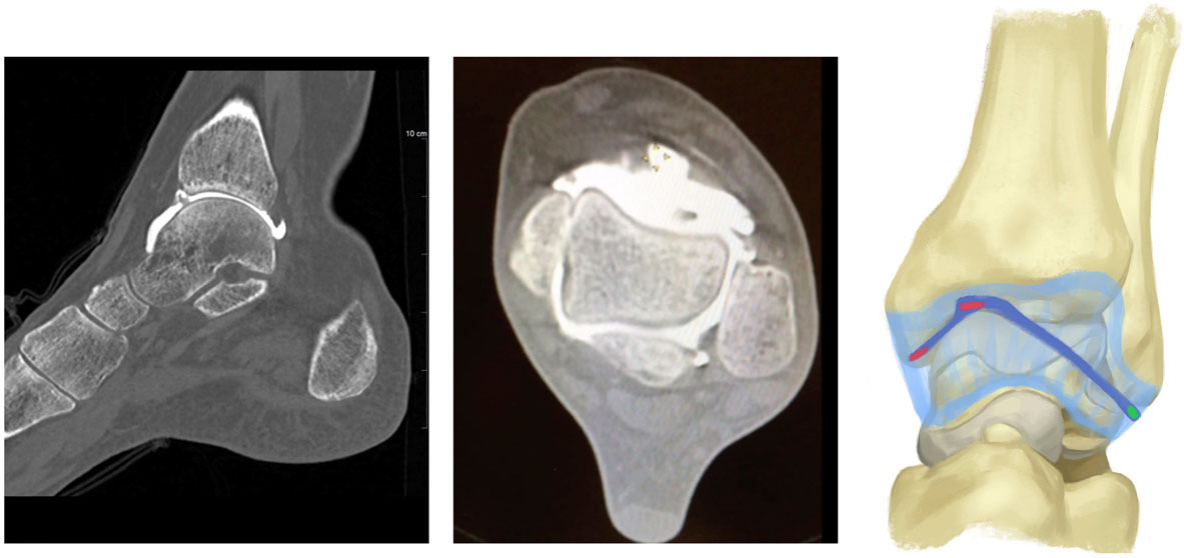
Sagittal and axial CTA views, and the shape of the fibrous band in patient 1

**Fig. 3:**
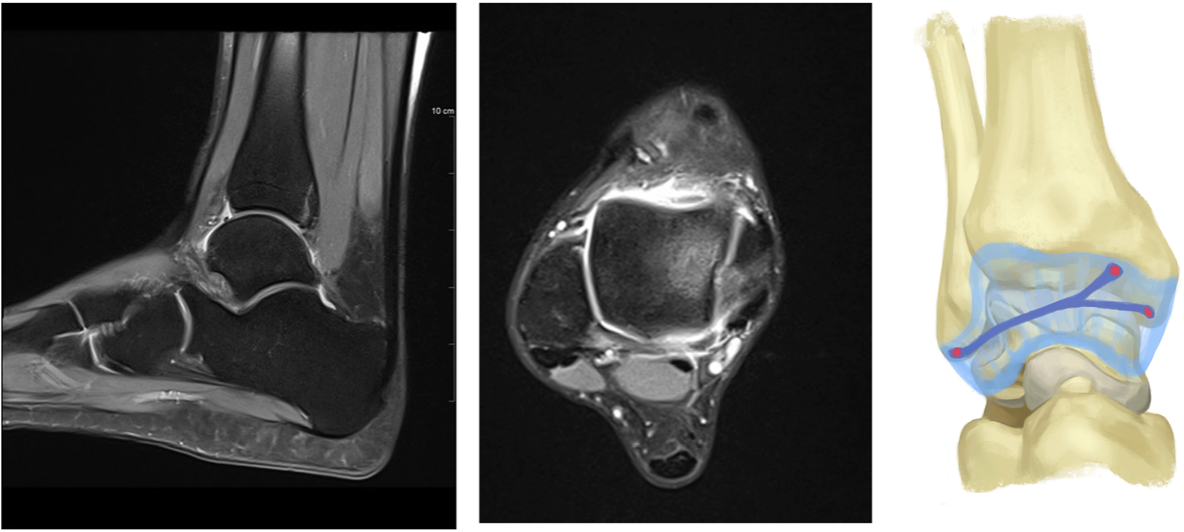
Sagittal and axial MRI views, and the shape of the fibrous band in patient 2

**Fig. 4:**
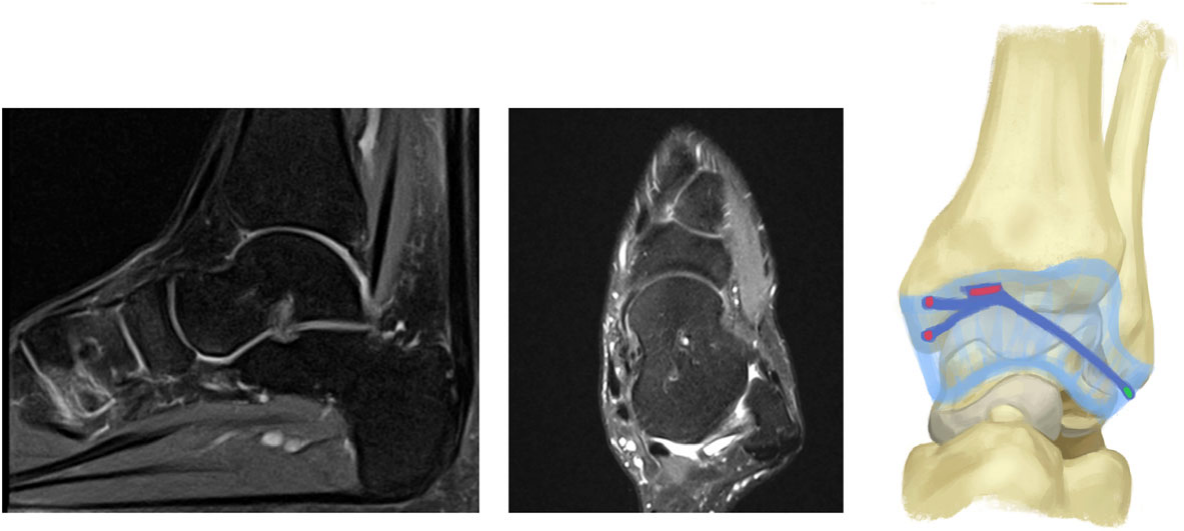
Sagittal and axial MRI views, and the shape of the fibrous band in patient 3

**Fig. 5:**
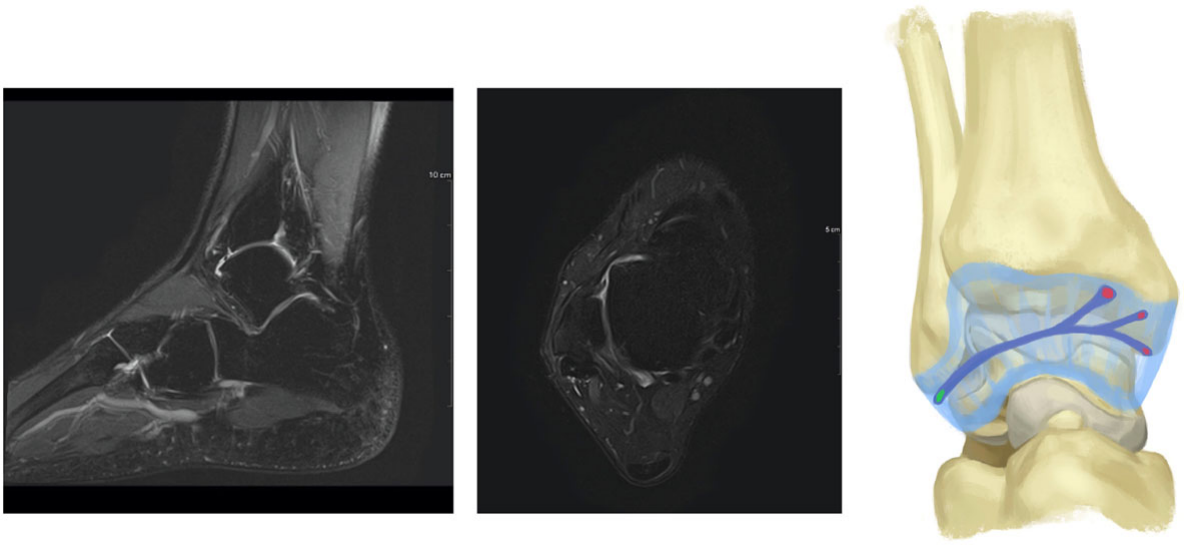
Sagittal and axial MRI views, and the shape of the fibrous band in patient 4

**Fig. 6:**
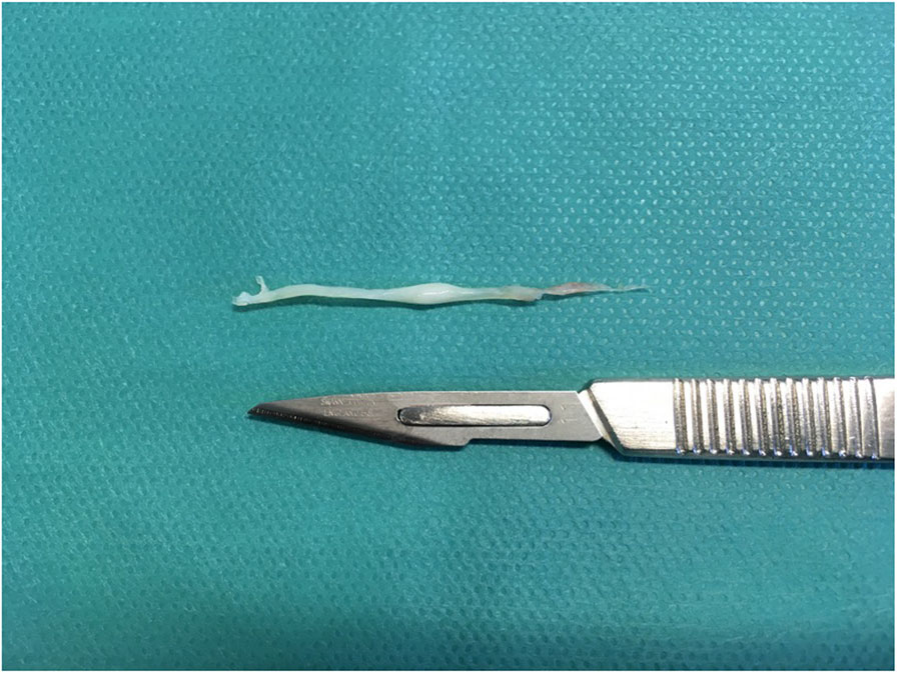
The fibrous band after resection

Arthroscopy was indicated for simple exploration and debridement in 2 patients that had persistent anterior ankle pain, while it was indicated to perform a Broström procedure in 2 patients to treat CAI. During arthroscopy, a chondral notch was discovered in 1 patient, on the superolateral edge of the talus, causing the fibrous band to jump over the notch. None of the four cases presented with a plica, nor with anterolateral impingement. One patient underwent conservative treatment for a superomedial osteochondral lesion, while the three other patients had no osteochondral lesions. The fibrous band was arthroscopically removed in all patients during exploration or a Brostöm procedure. The shape of the fibrous band was not consistent in every patient; in 1 patient it was V-shaped (Fig. 2), whereas in 3 patients it was Y-shaped (Figs. 3, 4 and 5). Histological examination revealed that the intra-articular band was fibrocartilaginous (Fig. 6).

At a minimum follow-up of 15 months, postoperative clinical scores were collected, including pain on visual analogue scale (pVAS), American Orthopaedic Foot & Ankle Society (AOFAS) [11] score, European Foot and Ankle Society score (EFAS) and EFAS sport [7], as well as overall subjective satisfaction from very unsatisfied to very satisfied.

At a follow-up of 22.3 ± 5.0 months (range, 15–26), all patients reported a decrease in pVAS (− 5.0 ± 2.6, range, 2–8), and an improvement in AOFAS (51.0 ± 17.7, range, 26–65), EFAS (14.5 ± 8.7, range, 6–23) and EFAS sport (8.0 ± 5.3, range, 2–10). Two patients rated their satisfaction with surgery as ‘very satisfied’, and 2 with ‘satisfied’.

## Discussion

The most important findings of this case report are the corroboration of an earlier discovery of unsual intra-articular fibrous bands in 4 ankles, with more detailed information for clinical and radiologic diagnosis, as well as outcomes of arthroscopic removal.

In the first 3 patients, no diagnosis of a fibrous band was made after MRI and CTA as it was overlooked because information on this pathology is very limited. In the last patient however, the radiologist and senior surgeon were able to detect it, and eventually confirm their findings by arthroscopic removal. In retrospect, the fibrous band was visible on all the available MRIs and CTAs, and could have been diagnosed. The authors of the present study can therefore confirm that this pathology can be diagnosed through MRI and CTA. In fact, in one patient, the intra-articular fibrous band was visible on both the MRI taken 7 months prior to arthroscopy and the MRI taken 10 years earlier, prior to extra-articular tenodesis. The appearance of the intra-articular fibrous band was identical on both MRIs, taken nearly 10 years apart, indicating that such anomalies could persist without structural modifications. Slavotinek et al. [9] corroborate that this pathology can be diagnosed by MRI, and presents itself as a filling defect within the ankle joint that is adherent at both end of the bone, articular or capsulo-ligamentous tissue.

During arthroscopy, a chondral notch was discovered in 1 patient on the superolateral edge of the talus. This notch is non-anatomic, and instead of originating from the trauma itself, friction of the fibrous band has possibly caused wear on the bone. This notch also made the fibrous band ‘jump’ in and out of notch, causing a feeling of ‘locking’. This phenomenon has previously been described in the knee after total knee arthroplasty, as it caused patellar snapping and jumping because of impingement [4, 13].

After arthroscopic removal of the band, histological examination revealed that it was fibrocartilaginous, confirming the findings of Valkering et al. [14]. In the present case report, all 4 patients had an ankle sprain between 6 months and 12 years prior to arthroscopy, suggesting that the fibrocartilage band could have formed secondary to traumatic haematoma and/or haemarthrosis. The intra-articular fibrous bands had a similar composition to cartilage tissue obtained by creating bone microperforations as described by Pridie [6], to stimulate osteochondral tissue formation. This procedure aims to promote tissue healing in knees with osteoarthristis, by inducing intra-articular bleeding, causing mesenchymal cells to multiply and differentiate into cartilage or bone. Pridie noted that this procedure creates fibrocartilage type II collagen, rather than hyaline cartilage [2], as confirmed by Valkering et al. [14].

To the authors’ knowledge, there is little published on intra-articular fibrous bands in the ankle, though there are reports of resembling plicae, also termed synovial tissue folds or ridges. Plicae have been reported in the knee to cause pain, clicking, instability, and reduce ROM. There are only few case reports of plicae in the ankle [3, 8, 10]. Somorjai et al. [10] reported on one ankle after a sprain where arthroscopic exploration revealed a white soft tissue structure originating from the anteromedial osteocartilaginous gutter, between the tibial plafond and medial malleolus. The structure resembling an intra-articular plica was arthroscopically removed, which relieved the pain. Highcock et al. [3] reported on one ankle with no previous sprains, where imaging revealed a ligament-like structure, under the tibialis anterior tendon, diagnosed as a plica. The plica was causing symptomatic “snapping” and pain, both of which were resolved by arthroscopic removal. Rosenbaum et al. [8] reported on two cases, one of which had multiple ankle sprains, that arthroscopic exploration revealed soft-tissue bands resembling intra-articular plicae. Both patients were relieved of pain after arthroscopic removal.

The present case report has a number of limitations, typical of investigations on rare or unknown conditions, including the small sample size and heterogeneity of preoperative imaging and clinical assessments, as well as differences in treatment modalities. Nevertheless, the present findings provide further evidence regarding the formation of intra-articular fibrous bands in the ankle, that often go undiagnosed due to their rarity and/or subtlety. The clincial relevance of these observations is that clinicians should beware of such foreign bodies in the ankle, particularly in patients with history of sprains, and consider arthroscopic removal in cases with persistent pain and/or functional impairment.

## Conclusion

This case report corroborates the findings of an earlier discovery of intra-articular fibrous bands in 4 ankles, with more detailed information for clinical and radiologic diagnosis, as well as outcomes of arthroscopic removal The clincial relevance of these observations is that clinicians should beware of such foreign bodies in the ankle, particularly in patients with history of sprains, and consider arthroscopic removal in cases with persistent pain and/or functional impairment.

## Data Availability

Data is available upon suitable request.

## Appendix

Patient 1: a 27-year old woman who reported a talar fracture after an ankle sprain in July 2018. In February 2019, she reported persistent pain at the anterolateral aspect of the medial malleolus. CTA revealed a loose body at the anterior aspect of the tibiotalar joint. In March 2019, during arthroscopy, a fibrous band was found between the malleoli, which was then succesfully resected.

Patient 2: a 24-year old woman who reported an ankle sprain during football in August 2017. In January 2019, she reported persistent pain at the anterolateral aspect of the medial malleolus, and a decreased ROM. MRI revealed ATFL thickening. In April 2019, during a Broström procedure, a fibrous band was found between the malleoli, which was then succesfully resected.

Patient 3: a 34-year old man who reported an ankle sprain in July 2018 during work. In August 2018, a MRI was performed with no particular pathology could be diagnosed. In January 2019, he reported persistent pain at the anterolateral aspect of the medial malleolus, and MRI revealed a torn ATFL, which required a Broström procedure. During this surgery a fibrous band was found between the malleoli, which was then succesfully resected.

Patient 4: a 50-year old man who reported an ankle sprain in April 2008 during work. In December 2009, he underwent an extra-articular tenodesis. In January 2019, he reported persistent pain at the anterolateral aspect of the medial malleolus, and a decreased ROM. In December 2019, during arthroscopy a fibrous band was found between the malleoli, which was then succesfully resected.
